# In Vitro and In Silico Evaluation of a Novel Multifunctional Cyclic Peptide with Antioxidant, Tyrosinase-Inhibitory, and Extracellular Matrix-Modulating Activities

**DOI:** 10.3390/ijms262210878

**Published:** 2025-11-09

**Authors:** Ga-Hyun Kim, Bo-Mi Kim

**Affiliations:** Department of Chemical Engineering, Wonkwang University, Iksan-si 54538, Jeollabuk-do, Republic of Korea; gahyun1214@wku.ac.kr

**Keywords:** cyclic peptide, DPPH radical scavenging activity, tyrosinase inhibition, MMP-1, procollagen, molecular docking

## Abstract

Peptides are notable cosmetic ingredients owing to their diverse biological activities and beneficial effects on skin health. Therefore, multifunctional peptides capable of simultaneously exerting antioxidant, whitening, and anti-wrinkle effects are highly desirable. In this study, a scalable and cost-effective chemical synthesis strategy was used for the rapid design and synthesis of linear peptide sequences with skin bioactivity using solid-phase peptide synthesis. Subsequently, liquid-phase peptide synthesis was used to enhance the proteolytic stability and develop a cyclic peptide, cyclic CYGSR (CR5), which was subjected to in vitro biological evaluation. CR5 showed high biocompatibility in water-soluble tetrazolium salt-1 (WST-1) assays, maintaining over 90% cell viability at concentrations up to 400 μg/mL. In the 2,2-Diphenyl-1-picrylhydrazy (DPPH) assay, CR5 exhibited strong antioxidant activity with 83.18% radical scavenging at 200 μg/mL. It also showed 97.79% tyrosinase inhibition at 800 μg/mL, confirming significant whitening potential. Moreover, CR5 inhibited matrix metalloproteinase-1 (MMP-1) expression by 73.55% and increased type I procollagen expression by 44.68% at 400 μg/mL, demonstrating its anti-wrinkle potential. Additionally, molecular docking and dynamic simulation demonstrated stable binding of the peptide to tyrosinase and MMP-1. Collectively, CR5 possesses multifunctional properties with excellent biocompatibility, highlighting its potential as a novel cosmetic active ingredient.

## 1. Introduction

The rapid advancement of medical technology has substantially extended human life expectancy, leading to an increasing societal interest in healthy aging, particularly skin aging. Age-related hyperpigmentation, oxidative stress, and loss of skin elasticity affect both aesthetic appearance and overall health, thereby heightening personal concerns and stimulating active scientific research on their mitigation [1,2]. Against the backdrop of global population aging, research on functional peptides for improving skin health has gained momentum in the cosmetics field.

A promising approach involves the discovery of bioactive peptides with antioxidant, whitening, and anti-wrinkle properties [3,4,5]. The major mechanisms of skin aging include the accumulation of oxidative stress, excessive melanin production, and extracellular matrix degradation, characterized by increased collagen breakdown and reduced procollagen synthesis. These processes act simultaneously and synergistically, leading to structural and functional deterioration of the skin. Specific bioactive functions exert beneficial effects, such as alleviation of oxidative stress, inhibition of melanin synthesis, and promotion of collagen expression, through mechanisms, including 2,2-Diphenyl-1-picrylhydrazy (DPPH) radical scavenging, tyrosinase inhibition, and suppression of matrix metalloproteinase-1 (MMP-1) expression. Such evaluations are widely used as indirect evidence of biological activities, such as antioxidant, whitening, and anti-wrinkle effects, and academically well recognized [6,7,8,9]. Within this context, peptide technology has been actively applied not only in biotechnology and pharmaceutical industries but also, more recently, in the cosmetics industry, where it is increasingly acknowledged as a next-generation platform for promoting skin health and preventing aging [10,11].

In addition to being auxiliary ingredients, peptides are being re-evaluated as core functional agents with intrinsic bioactivity and their applications continue to steadily expand [12,13,14]. Although linear peptides offer various advantages, concerns have been raised regarding their rapid degradation in the skin environment or within formulations. Cosmetic ingredients require high stability to minimize denaturation or decomposition during storage and use [15]. Experimental studies have confirmed the potential degradation of peptides in skin applications and cosmetic formulations, and factors, such as temperature, pH, and preservation conditions in aqueous systems critically influence peptide stability. These findings suggest that formulation stability is essential for the commercialization of peptide-based active ingredients [16,17].

To overcome these limitations, cyclic peptides have emerged as excellent candidates for cosmetic applications, offering greater stability and sustained efficacy. Their cyclic structure has multiple advantages, including enhanced resistance to proteolysis, improved structural robustness, and maintenance of a stable three-dimensional conformation. Cyclic peptides exhibit higher resistance to proteolytic enzymes and greater structural stability under aqueous conditions than those of their linear counterparts [18,19]. This implies that cyclic peptides may provide superior stability compared with that of linear peptides in cosmetic formulations that require long-term storage [14]. Furthermore, reports of the discovery of cyclic peptides with bioactive functions are increasing [20,21,22]. However, the synthesis of cyclic peptides remains challenging because of the demanding reaction conditions that can lead to cyclization failure, side reactions, and dimer formation [23,24]. Consequently, their production costs are higher than those of linear peptides and difficulties in scalability limit their economic feasibility for commercialization [25]. Current strategies offer limited efficiency in this regard; therefore, there is a need for optimized synthetic methods for the production of short cyclic peptides [26,27].

In the current research on skin aging, a variety of peptide materials have been reported. However, most are confined to single or dual functionalities, such as antioxidant activity, inhibition of melanin synthesis, or procollagen expression [28,29,30,31]. Skin aging is a multifactorial process involving multiple pathways; therefore, it is difficult to expect sufficient inhibitory effects from single-function materials alone. Therefore, an integrated multifunctional approach is required to develop novel peptide materials with practical applicability [32,33]. The development of biomaterials capable of simultaneously modulating multifactorial changes is a critical challenge in cosmetic and therapeutic science. From a commercial perspective, the demand for multifunctional ingredients in the cosmetic industry is steadily increasing. Currently, many products are formulated by combining different active ingredients, such as antioxidants, whitening agents, and anti-wrinkle agents, to achieve multiple benefits [32,34]. However, this strategy increases formulation complexity, introduces stability issues owing to potential interactions among the raw materials, and increases production costs [35]. Multifunctional ingredients that can simultaneously deliver antioxidant, whitening, and anti-wrinkle effects may effectively alleviate these issues. By fulfilling multiple functions with a single ingredient, such materials can simplify formulations, improve stability, and reduce costs, while also providing consumers with differentiated value through a “multi-effect.” Thus, the development of multifunctional peptides is important not only from an academic standpoint, but also from industrial and commercial perspectives, offering a clear direction for the creation of next-generation cosmetic actives [33,36].

In this study, the cyclic peptide, Cys-Tyr-Gly-Ser-Arg (CYGSR, CR5), was designed based on the fact that the properties of each amino acid are closely associated with skin bioactivity. Cysteine and tyrosine contribute to the antioxidant activity through radical scavenging mediated by thiol and phenolic groups, respectively. Glycine, a major structural amino acid in collagen, is linked to extracellular matrix (ECM) stability. Serine, a major component of natural moisturizing factors, possesses a water-binding capacity, and may contribute to skin hydration and barrier maintenance. Arginine promotes collagen synthesis and cellular activity, thereby supporting its anti-wrinkle efficacy. For these reasons, the CR5 sequence was rationally designed to exert multifunctional activities (antioxidant, whitening, and anti-wrinkle) simultaneously [37,38,39,40,41,42,43].

The designed sequence was synthesized via solid-phase peptide synthesis (SPPS) to produce a short linear peptide with antioxidant and skin-whitening properties [44]. Subsequently, a cyclization strategy based on liquid-phase peptide synthesis (LPPS) was used to shorten the production time and enable high-purity synthesis [45]. This approach suggests that CR5 has substantial potential not only for commercial applications but also as a bioactive material in both the pharmaceutical and cosmetic fields.

## 2. Results

### 2.1. Stucture of CR5

**Figure 1:**
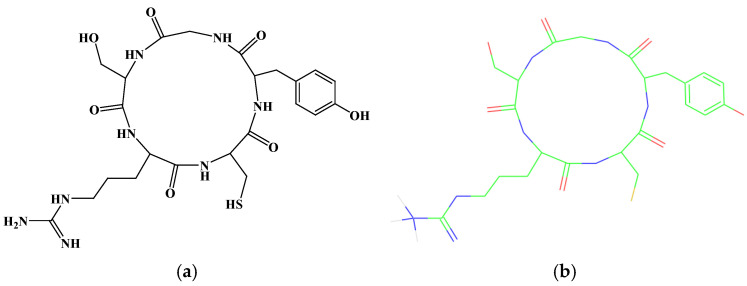
(**a**) The 2D structure of the cyclic peptide, Cys-Tyr-Gly-Ser-Arg (CR5). (**b**) The 3D structure of the cyclic peptide, CR5, predicted using molecular modeling (Color notation: The carbon skeleton is green, the oxygen/carbonyl group is red, and the nitrogen-related substituent is blue).

The cyclic peptide CYGSR was synthesized by cyclizing a linear pentapeptide composed of Cys–Tyr–Gly–Ser–Arg via intramolecular peptide bond formation. The 2D and 3D molecular structures of CR5 are shown in Figure 1a and Figure 1b, respectively.

### 2.2. Purity Analysis of CR5 by HPLC

**Figure 2:**
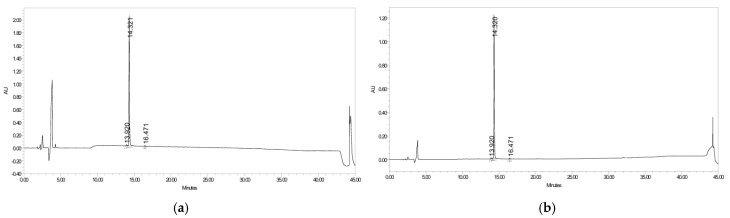
Analytical High-performance liquid chromatography (HPLC) chromatograms of CR5 measured at two detection wavelengths (X: retention time, Y: UV absorbance): (**a**) 210 nm and (**b**) 230 nm. A single sharp peak was observed in both chromatograms, confirming a purity of 98%.

An analytical High-performance liquid chromatography (HPLC) chromatogram of CR5 is shown in Figure 2. A single sharp peak was observed at a retention time of 14.32 min and no significant impurities were detected. The purity of CR5, calculated from the peak area integration at 210 (Figure 2a) and 230 nm (Figure 2b), was 98.0%, confirming that the peptide was obtained in a highly pure form. The original HPLC peak area integration report (chromatograms at 210 nm and 230 nm) as well as the exported time–absorbance XY data tables are provided in the Supplementary Materials (Supplementary Files S1 and S2).

### 2.3. Molecular Weight Analysis of CR5

**Figure 3:**
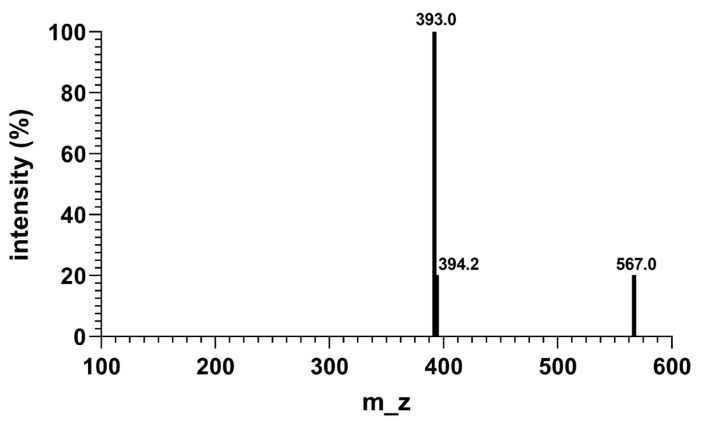
Matrix-assisted laser desorption/ionization time-of-flight mass spectrometry (MALDI-TOF MS) spectrum of CR5, showing the expected [M + H]^+^ ion peak.

The successful synthesis of CR5 was confirmed using Matrix-assisted laser desorption/ionization time-of-flight mass spectrometry (MALDI–TOF MS). MALDI-TOF MS analysis of CR5 revealed an [M + H]^+^ peak at *m*/*z* 567, corresponding to the theoretical molecular weight of the cyclic peptide (Figure 3). A prominent fragment peak at *m*/*z* 392.0 was interpreted as the complementary ion resulting from the neutral loss of NH_2_–Arg–COOH (MW: 174.20 g/mol), consistent with the expected fragmentation pattern. The adjacent peak at *m*/*z* 394 was attributed to an isotopic variant. Collectively, CR5 was successfully synthesized, as verified by MALDI–TOF MS analyses. The original MALDI graph (Supplementary File S3), are provided in the Supplementary Materials.

### 2.4. Cell Viability

**Figure 4:**
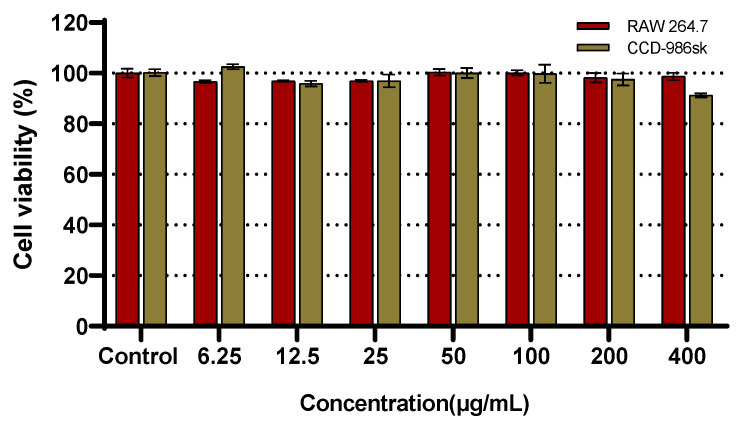
Effect of CR5 on the viability of RAW 264.7 and CCD-986sk cells, as determined using the water-soluble tetrazolium salt-1 (WST-1) assay.

In viable cells, mitochondrial dehydrogenases reduce water-soluble tetrazolium salt-1 (WST-1) to form the chromogenic compound, formazan, which is used to assess cell viability. The viability of both RAW 264.7 macrophages and CCD-986sk fibroblasts was evaluated using this assay. CR5 exhibited no cytotoxicity at concentrations below 400 μg/mL in either cell line, with the cells maintaining viability above 90% (Figure 4). These results indicate that the observed biological effects of CR5 in subsequent assays are not attributable to nonspecific cytotoxicity, but rather reflect its intrinsic functional activity under non-toxic conditions. The detailed cell viability values (WST-1 assay, CCD-986Sk and RAW 264.7) are provided in the Supplementary Materials (Supplementary File S4).

### 2.5. Antioxidant Activity Assay

**Figure 5:**
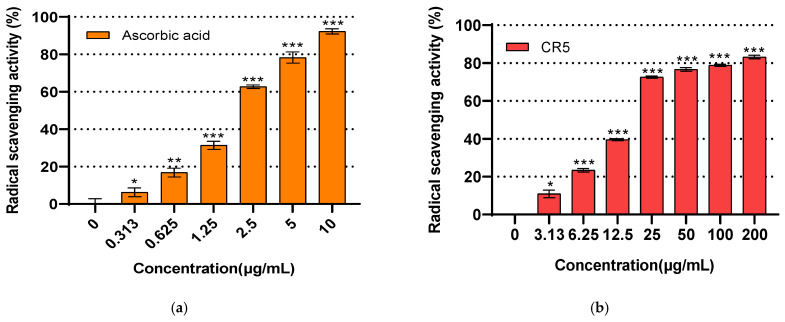

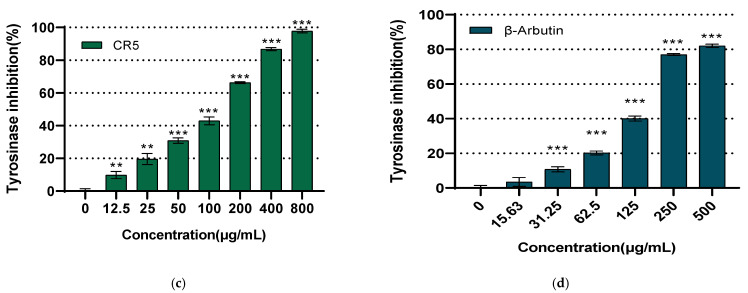
2,2-Diphenyl-1-picrylhydrazyl (DPPH) radical-scavenging activity. (**a**) Scavenging activity of CR5, IC_50_ = 16.92 ± 0.47 μg/mL (29.86 ± 0.83 μM, n = 3) and (**b**) positive control, ascorbic acid. Tyrosinase inhibitory activity: (**c**) inhibitory effect of CR5, IC_50_ = 104.2 ± 5.3 µg/mL (184.0 ± 9.4 µM, n = 3) and (**d**) the positive control, β-arbutin. (* *p* < 0.05, ** *p* < 0.01, *** *p* < 0.001).

The antioxidant activity of the synthesized CR5 was evaluated using the DPPH radical-scavenging assay, with ascorbic acid serving as a positive control (Figure 5a). CR5 exhibited concentration-dependent scavenging effects in the range of 3.13 to 200 μg/mL. At concentrations above 25 μg/mL, CR5 showed more than 50% scavenging activity. Specifically, at 200 μg/mL, a scavenging rate of 83.18% was observed, indicating that CR5 exerted a noticeable antioxidant effect (Figure 5b). CR5 exhibited statistically significant (*p* < 0.05) DPPH radical-scavenging activity. These findings suggest that CR5 may directly contribute to free radical neutralization, thereby mitigating oxidative stress, which is a key upstream trigger associated with ECM degradation and wrinkle formation.

### 2.6. Whitening Activity Assay

The whitening activity of the synthesized pentapeptide, CR5, was assessed based on its tyrosinase inhibitory effect. CR5 exhibited concentration-dependent inhibition over the range of 12.50 to 800 μg/mL. At the highest concentration tested, 800 μg/mL, CR5 demonstrated a strong whitening effect with an inhibition rate of 97.79% and the IC_50_ was determined to be 104.2 ± 5.3 μg/mL (Figure 5c). For comparison, β-arbutin, a widely used tyrosinase inhibitor, was also tested in the range of 31.25 to 500 μg/mL, and showed an IC_50_ of 160.12 μg/mL (Figure 5d). These results indicate that CR5 exhibited stronger tyrosinase inhibition than β-arbutin at comparable concentrations. CR5 exhibited statistically significant tyrosinase inhibitory activity (*p* < 0.05). This strong tyrosinase inhibitory effect suggests that CR5 can effectively inhibit melanin biosynthesis at the enzyme level, providing an electromotive basis for contributing to the whitening and hyperpigmentation inhibitory efficacy.

### 2.7. Type I Procollagen Expression Assay

**Figure 6:**
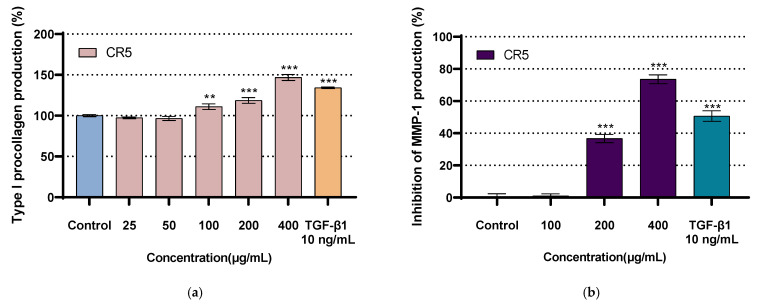
Effects of CR5 on type I procollagen production (**a**) and inhibition of UV-induced matrix metalloproteinase-1 (MMP-1) expression, IC_50_ = 261.3 ± 12.0 µg/mL (461.9 ± 21.2 µM, n = 5) (**b**) in human fibroblasts (** *p* < 0.01, *** *p* < 0.001).

The effect of CR5 on type I procollagen production in human fibroblasts is shown in Figure 6a. TGF-β1 was used as a positive control. CR5 significantly (*p* < 0.05) promoted type I procollagen expression in a concentration-dependent manner within the range of 100–400 μg/mL. Notably, treatment with 400 μg/mL of CR5 resulted in an approximately 44.68% increase in type I procollagen expression compared with that in the untreated control group, and was approximately 30.12% higher than that with the positive control, TGF-β1. CR5 exhibited statistically significant (*p* < 0.05) type I procollagen expression activity. These results suggest that CR5 may directly induce the mechanical effects associated with wrinkle relief and skin elasticity retention by promoting the synthesis of ECM components.

### 2.8. Inhibition of MMP-1 Expression Assay

The effect of CR5 on UV-induced MMP-1 production in human dermal fibroblasts is shown in Figure 6b. TGF-β1 was used as a positive control. Compared with the UV-treated control, CR5 significantly reduced MMP-1 expression at concentrations of 200–400 μg/mL (*p* < 0.05). In particular, treatment with 400 μg/mL of CR5 resulted in a 73.55% inhibition of MMP-1 expression. CR5 significantly (*p* < 0.05) inhibited MMP-1 expression (IC_50_ = 261.3 ± 12.0 µg/mL). This MMP-1 inhibitory effect suggests that CR5 may mechanically contribute to the inhibition of wrinkle production by inhibiting collagen degradation and thus mitigating ECM collapse.

### 2.9. Molecular Docking of CR5 with Target Enzymes (Tyrosinase and MMP-1)

**Figure 7:**
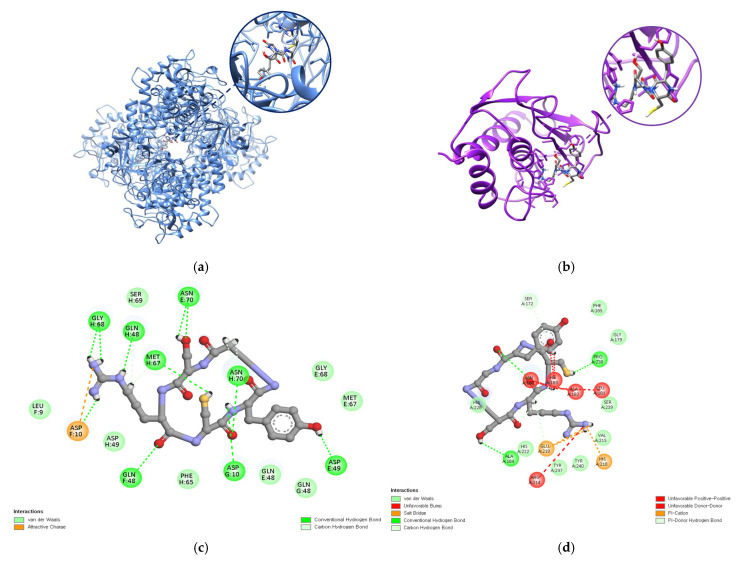
(**a**) 3D visualization of the molecular docking of CR5 with tyrosinase (PDB ID: 2Y9X) (**b**) 3D visualization of the molecular docking of CR5 with MMP-1 (PDB ID: 1HFC). (**c**) 2D visualization of the molecular docking of CR5 with tyrosinase (**d**) 2D visualization of the molecular docking of CR5 with MMP-1.

Molecular docking simulations were performed using the three-dimensional structures of tyrosinase (PDB ID: 2Y9X) and MMP-1 (PDB ID: 1HFC) to evaluate the binding affinity of the cyclic peptide, CR5. According to the docking results, CR5 exhibited a strong binding affinity to the active site of the tyrosinase-like protein, with a MolDock Score of −110.023 kcal/mol and hydrogen bond contribution of −14.81 kcal/mol. The substantial hydrogen bond energy suggests the presence of specific directional interactions. These findings indicate that CR5 may inhibit the expression or activity of tyrosinase, highlighting its potential as a functional peptide with whitening and antioxidant properties. The three-dimensional visualization is presented in Figure 7a.

In addition, CR5 showed stable binding to the active site of MMP-1, with a MolDock Score of −99.64 kcal/mol and a hydrogen bond contribution of −9.20 kcal/mol, indicating a strong binding affinity and possibility of specific interactions (Table 1). This suggests that CR5 may act as a bioactive peptide capable of inhibiting MMP-1 activity. Further structural optimization and biochemical validation are required to confirm the inhibitory potential of CR5. The three-dimensional visualization is presented in Figure 7b.

The 2D molecular docking analysis revealed that CR5 bound stably to the active site of tyrosinase (PDB ID: 2Y9X). Molecular docking of the cyclic peptide CR5 with tyrosinase (PDB ID: 2Y9X) revealed a stable binding conformation within the catalytic pocket of the enzyme. CR5 established multiple hydrogen bonds with key residues, including Gln48, Gln49, Gly68, Asn70, and Asp10, and exhibited an electrostatic interaction with Asp10. These interactions were further stabilized by van der Waals contacts with surrounding residues such as Met67, Phe65, and Leu9, thereby enhancing the stability of the complex. This interaction profile suggests that CR5 can effectively occupy the catalytic pocket and potentially inhibit tyrosinase activity (Figure 7).

In addition, CR5 formed several non-covalent interactions within the active site of MMP-1 (PDB ID: 1HFC). Specifically, hydrogen bonds were observed with His218, Tyr240, and Gly179, along with π–π stacking with Tyr237 and an electrostatic interaction with Arg214. Furthermore, van der Waals contacts with residues such as Ser172, Pro238, Val215, and Phe185 further stabilized the binding conformation. Notably, the involvement of Cys, Ser, and Arg residues in the interaction network supports their putative contributions to the inhibition of both tyrosinase and MMP-1. Collectively, these docking results emphasize the multifunctional inhibitory potential of CR5 against key enzymes implicated in skin aging.

## 3. Discussion

In this study, the cyclic pentapeptide, CR5, exhibited multiple bioactivities associated with skin aging. Specifically, CR5 demonstrated statistically significant (*p* < 0.05) DPPH radical-scavenging activity, tyrosinase inhibitory activity, promotion of type I procollagen expression, and suppression of UV-induced MMP-1 expression. These findings suggest that CR5 has potential as a multifunctional ingredient in cosmetics. Molecular docking simulations using the crystal structures of MMP-1 (PDB ID: 2Y9X) and tyrosinase (PDB ID: 1HFC) predicted the potential binding modes of CR5 to its target proteins. These computational findings, together with the in vitro results, provide supportive evidence for the multifunctional activities of CR5 and further suggest plausible molecular mechanisms underlying its antioxidant, skin-whitening, and anti-wrinkle effects.

Most previously reported peptides have been restricted to single or, at best, dual functionalities, such as antioxidant activity, inhibition of melanin synthesis, or stimulation of collagen production. In contrast, CR5 exhibited a distinctive profile, exerting integrated inhibitory effects on multiple pathways involved in skin aging. Based on previous studies, the cyclic structure was expected to provide stereochemical stability and resistance against enzymatic degradation [19,46,47,48]. In addition, the cysteine residue may contribute to antioxidant activity through its reducing properties, whereas amino acid residues, such as serine and arginine are presumed to be involved in the regulation of collagen synthesis or inhibition of tyrosinase activity [37,38,39,40,41,42,43,49]. The multifunctionality of CR5 has important implications from academic and industrial perspectives. Academically, it is valuable because it presents a peptide candidate capable of simultaneously modulating the major factors involved in skin aging, including oxidative stress, melanin synthesis, and ECM degradation. From an industrial perspective, multifunctional cosmetic formulations require a combination of antioxidants, whitening agents, and anti-wrinkle ingredients [32,33]. CR5 has the potential to replace complex formulations with a single peptide, thereby improving formulation stability and reducing production costs.

CR5 developed in this study exhibited overall superior efficacy compared with previously reported cases. In terms of DPPH radical scavenging, earlier studies have reported IC_50_ values in the range of approximately 35–1680 µg/mL, and within this comparative range, CR5 demonstrated relatively potent radical-scavenging activity (IC_50_ = 16.92 ± 0.47 μg/mL) [50,51,52]. In addition, previous studies have shown that the IC_50_ for tyrosinase inhibition was approximately 146–172 mg/mL, further supporting that CR5 has markedly stronger inhibitory capacity compared with reported values (IC_50_ = 104.2 ± 5.3 µg/mL) [53,54]. The inhibitory effect on MMP-1 expression and the increase in type I procollagen were evaluated using TGF-β1 as a positive control, and multiple previous reports support the role of TGF-β1 as a positive regulator that suppresses MMP-1 and promotes procollagen synthesis. Under the same experimental conditions, CR5 (400 µg/mL) exhibited 73.55% inhibition of MMP-1 and a 44.68% increase in type I procollagen, indicating a stronger efficacy than TGF-β1. Therefore, CR5 can be considered a promising peptide candidate with potential applicability as a functional cosmetic ingredient, particularly owing to its MMP-1 inhibitory activity and ECM-preserving effect [55,56].

However, this study had several limitations. First, the findings were restricted to in vitro experiments and further validation is required to demonstrate the applicability of CR5 in cosmetic formulations. Although molecular docking provides predictive evidence for the potential of CR5 to inhibit specific enzymes, detailed investigations into intracellular signaling pathways and peptide–protein interactions remain insufficient. Further assessments are necessary to evaluate skin permeability, long-term stability, and bioavailability for practical applications. From a molecular docking perspective, the present docking analysis was limited to the evaluation of MolDock Score and Rerank Score-based fitting quality [57]. Thus, in future work, a more comprehensive validation framework incorporating additional scoring functions and validation metrics (e.g., RMSF or per-residue interaction energy) will be necessary to further substantiate the docking reliability.

Therefore, future research should include in vivo validation using animal models, along with mechanistic studies of signaling pathways and peptide–protein interactions. Furthermore, structure–activity relationship studies using CR5 analogs are necessary to elucidate the molecular basis of their multifunctional properties. Additional investigations will provide critical evidence for the development of CR5 as a novel multifunctional cosmetic and therapeutic peptide targeting skin aging.

## 4. Materials and Methods

### 4.1. Materials

#### 4.1.1. Cell Lines

Human dermal fibroblasts (CCD-986sk) and mouse macrophages (RAW 264.7) were obtained from the Cell Line Bank of Korea (KCLB, Seoul, Republic of Korea).

#### 4.1.2. Assay Kits

Cell viability was measured using an EZ-CYTOX kit (EZ-1000; Daeil Lab Service, Seoul, Republic of Korea). Protein concentrations were determined using the Pierce BCA Protein Assay Kit (23227; Thermo Fisher Scientific, Waltham, MA, USA). Type I procollagen levels were quantified using the Procollagen Type I C-Peptide EIA Kit (MK101; Takara Bio, Shiga, Japan). The secretion of MMP-1 was measured using a Human Total MMP-1 Quantikine enzyme-linked immunosorbent assay (ELISA) Kit (DMP100, R&D Systems, Minneapolis, MN, USA). Protein was quantified using an Albumin Standard (23209, Thermo Fisher Scientific) as a reference.

#### 4.1.3. Chemicals

All amino acids used in this study were purchased from GL Biochem (Shanghai, China). The reagents for peptide synthesis and other chemical analyses were obtained from Sigma-Aldrich (St. Louis, MO, USA) or Tokyo Chemical Industry (Tokyo, Japan).

#### 4.1.4. Instruments

HPLC analysis of the synthesized peptides was performed using a Waters e2695 separation module equipped with a 2998 photodiode array (PDA) detector (Waters Corp., Milford, MA, USA). Data acquisition and processing were performed using the Empower software3 (Waters Corp.). Preparative purification was performed using a Waters 2545 Quaternary Gradient Module equipped with a 2998 PDA detector (Waters Corp.). A cold laboratory chamber (JSS-700C, JS Research Inc., Gongju, Republic of Korea), microplate multi-reader (Synergy HTX, BioTek Instruments, Winooski, VT, USA), and microbalance (ARG4202, OHAUS Corp., Parsippany, NJ, USA) were also used in this study.

### 4.2. Methods

#### 4.2.1. Synthesis of CYGSR Using Fmoc-Chemistry

The CYGSR peptide used in this study was synthesized using SPPS. The 2-chlorotrityl chloride resin (0.50 g, 1.00 mmol/g) was swollen in dichloromethane (DCM, 5.80 mL) for 30 min. After swelling, the solvent was removed under reduced pressure and the resin was washed twice with DCM. The initial coupling of the first amino acid was performed with Fmoc-Arg(Pbf)-OH (2.00 eq, 0.60 g) and N,N-diisopropylethylamine (DIEA, 5.00 eq, 0.40 mL) for 4 h, followed by capping of unreacted sites with DCM: Methanol: DIEA (15:3:2, *v*/*v*) for 10 min.

For sequential coupling, Fmoc-Ser(tBu)-OH (2.00 eq, 0.40 g) was activated with 1-hydroxybenzotriazole (HOBt, 1.40 eq, 0.20 g), DIEA (5.00 eq, 0.40 mL), and N,N′-diisopropylcarbodiimide (DIC, 3.00 eq, 0.20 mL) in N,N-dimethylformamide (DMF, 4.00 mL), and the mixture was reacted with the resin for 4 h. Completion of the coupling reaction was confirmed using the Kaiser test. Based on the test results, double coupling or capping of the unreacted amines (DMF: Acetic anhydride: DIEA, 8:1:1, *v*/*v*) was performed as required.

Amino acid coupling was performed after Fmoc deprotection using 20% piperidine in DMF. The third [Fmoc-Gly-OH (2.00 eq, 0.30 g)], fourth [Fmoc-Tyr(tBu)-OH (2.00 eq, 0.50 g)], and fifth [Fmoc-Cys(Trt)-OH (2.00 eq, 0.60 g)] amino acids were sequentially coupled following the same procedure as described for the initial coupling.

#### 4.2.2. Cleavage of CYGSR

To cleave the synthesized CYGSR from the resin, 12 mL of a cleavage cocktail consisting of trifluoroacetic acid (TFA), triisopropylsilane, and H_2_O (95:2.5:2.5, *v*/*v*/*v*) was added to the reaction vessel and then stirred for 1 h. The mixture was filtered, and the filtrate was precipitated with 66 mL of cold isopropyl ether to yield a white solid.

#### 4.2.3. Cyclization of CYGSR

The linear peptide, CYGSR, was cyclized through the formation of an intramolecular amide bond between the N-terminal amine and C-terminal carboxylic acid. Lyophilized CYGSR (1.00 mmol) was dissolved in DMF to a final concentration of 1.00 mM. HOBt (2.50 eq), N-ethyl-N′-(3-dimethylaminopropyl)carbodiimide hydrochloride (EDC·HCl, 2.50 eq), and DIPEA (5.00 eq) were sequentially added with stirring at 0 °C. The reaction mixture was then stirred under nitrogen atmosphere at room temperature, and aliquots were taken at 4 h intervals for monitoring using HPLC. After 12 h, the reaction was terminated and the cyclic peptide, CYGSR (CR5), was obtained by precipitation induced by the addition of cold distilled water. The product was extracted three times with ethyl acetate, and the combined organic layers were dried over anhydrous sodium sulfate (Na_2_SO_4_) and concentrated under reduced pressure. The crude peptide was purified using reversed-phase HPLC.

#### 4.2.4. HPLC Purification and Analysis of CR5

Purification was performed using a preparative HPLC system (Waters 2545 Quaternary Gradient Module with 2998 PDA) after dissolving 0.05 g of the crude peptide in 5 mL of 0.10% TFA in water. A YMC-Pack ODS-A-HG C18 reversed-phase column (YMC, Kyoto, Japan) was used. The mobile phase consisted of solvent A (0.10% TFA in water) and B (0.10% TFA in acetonitrile), and separation was performed under the gradient conditions listed in Table 2.

The obtained peptide was concentrated by removing acetonitrile using a rotary evaporator and subsequently dried using lyophilization to yield a powder.

The analysis of peptide, CR5, was performed using the same instrument as that used for purification, with an XBridge C18 column (Waters). The detailed analytical conditions are listed in Table 3.

Detection wavelengths were monitored at 210, 230 nm, a region that strongly detects the amide binding of peptides.

#### 4.2.5. MALDI-TOF Mass Spectrometry (MS) Analysis of CR5

Since CR5 is a newly synthesized cyclic pentapeptide, verifying that the measured *m*/*z* matches the theoretical monoisotopic mass (566.64 Da) is a primary step to confirm successful cyclization and molecular identity prior to biological evaluation. MALDI-TOF MS was performed using an Autoflex maX MALDI-TOF/TOF mass spectrometer equipped with a SmartBeam II laser (355 nm; Bruker Daltonics; High-Tech Materials Analysis Core Facility, GNU, Jinju, Republic of Korea). A saturated solution of α-cyano-4-hydroxycinnamic acid in TA50 (50:50, *v*/*v*, acetonitrile: 0.1% TFA in water) was used as the matrix. The peptide samples were dissolved in 100 μL of 0.1% TFA, mixed with the matrix solution at a 1:1 ratio, spotted onto a MALDI target plate, air-dried, and subjected to analysis. The measurements were carried out in the positive ion reflectron mode under the following instrumental conditions: laser power, 35–40%; laser frequency, 2000 Hz; ion source 1, 19 kV; ion source 2, 16.8 kV; and pulsed ion extraction, 110 ns. The MS spectra were acquired over a detection range of 300–4000 Da using 500 accumulated laser shots. Data processing was performed using the FlexAnalysis software version 3.4 (Bruker Daltonics). External calibration was conducted using a Peptide Calibration Standard (Bruker Daltonics), including Angiotensin II ([M + H]^+^ = 1046.5418), Angiotensin I ([M + H]^+^ = 1296.6848), Substance P ([M + H]^+^ = 1347.7354), Bombesin ([M + H]^+^ = 1619.8223), ACTH clip 1–17 ([M + H]^+^ = 2093.0862), ACTH clip 18–39 ([M + H]^+^ = 2465.1983), and Somatostatin 28 ([M + H]^+^ = 3147.4710).

#### 4.2.6. Cell Culture

The mouse macrophage cell line RAW 264.7 and human dermal fibroblast cell line, CCD-986sk, were cultured in Dulbecco’s Modified Eagle’s medium (DMEM; high glucose) supplemented with 10% fetal bovine serum (FBS) and 1% penicillin–streptomycin. Cells were maintained in 100 mm culture dishes in a 5% CO_2_ incubator at 37 °C. Upon reaching confluence, the cells were sub-cultured using a cell scraper to maintain cell proliferation. A human-derived fibroblast cell line was cultured in Iscove’s Modified Dulbecco’s medium (IMDM) supplemented with 10% FBS and 1% penicillin–streptomycin in 150 mm culture dishes under the same incubation conditions. Upon confluence, the cells were passaged using mild trypsinization.

#### 4.2.7. Cell Viability Assay in RAW 264.7 and CCD-986sk Cells

In viable cells, mitochondrial dehydrogenases reduce the water-soluble tetrazolium salt (water-soluble tetrazolium salt, WST-1) to form a chromogenic formazan product, which is quantified to assess cell viability. RAW 264.7 cells were seeded at a density of 1 × 10^4^ cells per well in 96-well culture plates and incubated for 24 h in DMEM supplemented with 10% FBS and 1% penicillin–streptomycin. After incubation, the medium was replaced with fresh DMEM containing the test samples at various concentrations, followed by a 24 h treatment period. At the end of the treatment, the EZ-CYTOX reagent (DoGenBio, Seoul, Republic of Korea) was added to each well and plates were incubated according to the manufacturer’s instructions. Cell viability was determined by measuring the absorbance at 450.00 nm using a microplate reader. The percentage of viable cells was calculated using Equation (1).

CCD-986sk human dermal fibroblasts were seeded at a density of 3 × 10^3^ cells per well in 96-well plates and cultured for 24 h in IMDM supplemented with 10% FBS and 1% penicillin–streptomycin. The medium was then replaced with serum-free IMDM (1% penicillin–streptomycin) and cells were serum-starved for at least 6 h. After starvation, the cells were harvested. The test compounds were dissolved in distilled water to prepare stock solutions and diluted to final concentrations using serum-free IMDM (1% penicillin–streptomycin). The culture medium was carefully aspirated and replaced with sample-containing medium. The cells were then incubated under standard conditions for 48 h. After incubation, each well was treated with serum-free IMDM containing 10% EZ-CYTOX for 2 h. Cell viability was quantified by measuring the absorbance at 450.00 nm using a microplate reader and calculated according to Equation (1).

Cell viability (%):

(1) $$\left(\frac{A_{sample}}{A_{control}}\right)\times 100$$

#### 4.2.8. DPPH Radical-Scavenging Assay

To assess the antioxidant activity of the test samples, serial dilutions of the synthesized peptides were prepared, using ascorbic acid as a positive control. Each reaction mixture consisted of 12.5 μL of the sample solution, 50 μL of ethanol, and 62.5 μL of 0.10 mM DPPH solution. The mixtures were incubated for 30 min at 4 °C in the dark. After the reaction, the absorbance was measured at 520 nm using a multimode microplate reader. The free radical-scavenging activity (%) was calculated using Equation (2).

Radical-scavenging activity (%):

(2) $$100-\left(\frac{b-b^{\prime }}{a-a^{\prime }}\right)\times 100$$

a: Absorbance after reaction of stock solution.

b: Absorbance after reaction of sample solution.

a′, b′: Absorbance measured by substituting the buffer for 0.10 mM DPPH.

#### 4.2.9. Tyrosinase Inhibition Assay

To assess tyrosinase inhibitory activity, serial dilutions of the synthesized peptide and the positive control, β-arbutin, were prepared. In a 96-well plate, 10 μL of sample solution was mixed with 110 μL of 0.10 M sodium phosphate buffer (pH 6.5), followed by the addition of 10 μL of mushroom tyrosinase solution (1500 U/mL) and 20 μL of 1.50 mM L-tyrosine. The reaction mixture was then incubated at 37 °C for 10 min. The absorbance was measured at an appropriate wavelength (475 nm) using a microplate reader and the percentage of tyrosinase inhibition was calculated using Equation (3).

Tyrosinase inhibition (%):

(3) $$100-\left(\frac{b-b^{\prime }}{a-a^{\prime }}\right)\times 100$$

a: Absorbance after reaction of stock solution.

b: Absorbance after reaction of sample solution.

a′, b′: Absorbance measured by substituting the buffer for mushroom tyrosinase.

#### 4.2.10. MMP-1 Expression Inhibition Assay

To evaluate the inhibitory effect on MMP-1 expression, TGF-β1 (10 ng/mL) was used as the positive control. CCD-986sk cells were seeded into 24-well culture plates at a density of 1.5 × 10^4^ cells/well and cultured for 24 h in IMDM supplemented with 10% FBS and 1% penicillin–streptomycin. The cells were then incubated in serum-free IMDM for 6 h to induce starvation, washed with 1 mL of DPBS, and irradiated with UV (5 J/cm^2^). UV irradiation (5 J/cm^2^) was performed under non-cytotoxic conditions previously reported to induce MMP-1 expression [58,59]. The test samples were diluted in serum-free IMDM, applied to the cells, and incubated for 48 h. After 48 h, the culture supernatants were collected and analyzed using ELISA, and the inhibition rate of MMP-1 expression was calculated according to Equation (4).

MMP-1 Expression Inhibition (%):

(4) $$100-\left(\frac{b}{a}\right)\times 100$$

a: Expression of the corrected untreated group.

b: Expression of the corrected sample treatment group.

#### 4.2.11. Type I Procollagen Expression Stimulation Assay

ELISA was performed using a Type I Procollagen C-Peptide EIA Kit. Antibody-POD Conjugate solution (100 μL/well) was dispensed into the provided well strips, followed by the addition of 20 μL/well of standard solution or sample. After mixing, the plates were incubated at 37 °C for 3 h. Following incubation, the supernatants were discarded and wells were washed four times with wash buffer. Subsequently, 100 μL/well of Substrate Solution (TMBZ) was added and incubated at room temperature for 15 min, after which 100 μL/well of stop solution was added to terminate the reaction. Absorbance was measured at 450 nm using a multiplate reader. The expression levels of type I procollagen were compared and stimulation rate was calculated according to Equation (5).

Type I Procollagen Expression Promotion Rate (%):

(5) $$100-\left(\frac{b}{a}\right)\times 100$$

a: Expression of the corrected untreated group.

b: Expression of the corrected sample treatment group.

#### 4.2.12. Protein Content Analysis

Protein content was measured using a Pierce BCA Protein Assay kit. After cell culture, 20 μL of the culture medium was mixed with 200 μL of the Pierce BCA Protein Assay reagent mixture and incubated at 37 °C for 30 min. The absorbance was then measured at 570 nm. The protein concentration was determined using an Albumin Standard as a reference.

#### 4.2.13. Data Analysis and Statistical Processing

Statistical significance of all experimental results was evaluated using GraphPad Prism 8 software (GraphPad Software, San Diego, CA, USA). One-way ANOVA and Dunnett post hoc test were performed, and differences were considered statistically significant at *p* < 0.05.

#### 4.2.14. Molecular Docking Simulation

The three-dimensional structure of the cyclic peptide, CR5, was generated using Chem3D Ultra 8.0. The crystal structures of the target enzymes, human MMP-1 (PDB ID:1HFC) and mushroom tyrosinase (PDB ID: 2Y9X), were obtained from the RCSB Protein Data Bank (https://www.rcsb.org/structure/2Y9X (accessed on 29 September 2025) and https://www.rcsb.org/structure/1HFC (accessed on 29 September 2025)). Molecular docking simulations were performed using Molegro Virtual Docker 6.0 to predict the binding mode between the peptide and target proteins. The docking results were analyzed based on the binding-affinity scores and key amino acid residues involved in the interactions. Additionally, the two-dimensional visualization of the docking interactions was generated using Discovery Studio Visualizer, whereas the three-dimensional visualization was performed with Chimera version 1.19.

The docking parameters were defined as follows. For tyrosinase (PDB ID: 2Y9X) and MMP-1 (PDB ID: 1HFC), the corresponding metal ions were retained in their crystallographic positions (Cu(II) for tyrosinase; Zn(II)/Ca(II) for MMP-1). Bulk water molecules were removed except for structural waters involved in metal coordination. The grid box was centered at the active site (tyrosinase: X = −5.39, Y = −0.97, Z = −67.76 Å; MMP-1: X = 14.54, Y = 30.08, Z = 24.38 Å) with a radius of 15 Å. The protein structures were treated as rigid receptors, and docking was performed with fixed side chains. All ligands were prepared in their most probable protonation states at pH 7.4 to reflect physiological conditions. The reliability of the docking data was evaluated based on the MolDock Score and the Rerank Score generated by the MVD program. These parameters are energy-based scoring functions that quantitatively reflect the binding affinity between a ligand and its target receptor, where lower energy values correspond to a more thermodynamically favorable and stable binding conformation. Binding affinity scores were expressed in kcal/mol.

## Figures and Tables

**Table 1:** Molecular docking results of the cyclic peptide CR5 with tyrosinase-like protein (PDB ID: 2Y9X) and MMP-1 (PDB ID: 1HFC).

	MolDock Score (kcal/mol)	H-Bond Energy (kcal/mol)	Rerank Score
PDB ID: 2Y9X	−110.023	−14.8095	−72.9746
PDB ID: 1HFC	−99.6438	−9.19535	27.7123

**Table 2:** HPLC purification condition.

**Column**	YMC-Pack ODS-A-HG (250.00 × 20.00 mm I.D., 10.00 µm, 30.00 nm)	
**Mobile Phase**	Solvent A: H_2_O + 0.10% TFA Solvent B: Acetonitrile + 0.10% TFA	
**Time (min)**	**Solvent A (%)**	**Solvent B (%)**
0.0	95	5
10.0	95	5
45.0	50	50
50.0	50	50
50.1	0	100
51.0	0	100
51.1	95	5
55.0	95	5
**Flow Rate**	20.00 mL/min	
**Detection Wavelength**	210.00 nm or 230.00 nm	
**Injection Volume**	1.00~5.00 mL	
**Run Time**	55 min	

**Table 3:** HPLC analytical conditions.

**Column**	XBridge C18 column (4.60 × 250.00 mm, 5.00 μm)	
**Mobile Phase**	Solvent A: H_2_O + 0.10% TFA Solvent B: Acetonitrile + 0.10% TFA	
**Time (min)**	**Solvent A (%)**	**Solvent B (%)**
0.0	95	5
10.0	95	5
35.0	35	65
40.0	35	65
40.1	0	100
41.0	0	100
41.1	95	5
45.0	95	5
**Flow Rate**	1.00 mL/min	
**Detection Wavelength**	210.00 nm or 230.00 nm	
**Injection Volume**	20.00~100.00 μL	
**Run Time**	45 min	

## Data Availability

The data presented in this study are available on request from the corresponding author. The data are not publicly available due to [The data presented in this study are available on request from the corresponding author. The data are not publicly available due to confidentiality and ongoing further development].

## Supplementary Materials

The following supporting information can be downloaded at: https://www.mdpi.com/article/10.3390/ijms262210878/s1.

## Abbreviations

The following abbreviations are used in this manuscript:

CR5	Cys-Tyr-Gly-Ser-Arg
DCM	Dichloromethane
DIEA	N,N-diisopropylethylamine
DMF	N,N-dimethylformamide
DMEM	Dulbecco’s Modified Eagle’s medium
ECM	Extracellular matrix
FBS	Fetal bovine serum
HOBt	1-hydroxybenzotriazole
DIC	N,N′-diisopropylcarbodiimide
IMDM	Iscove’s Modified Dulbecco’s medium
MALDI–TOF	Matrix-assisted laser desorption/ionization time-of-flight
MS	Mass spectrometry
PDA	Photodiode array
SPPS	Solid-phase peptide synthesis
LPPS	Liquid-phase peptide synthesis
TFA	Trifluoroacetic acid
