# Supplementary material for: In Vitro and In Silico Evaluation of a Novel Multifunctional Cyclic Peptide with Antioxidant, Tyrosinase-Inhibitory, and Extracellular Matrix-Modulating Activities
Source: Int J Mol Sci. 2025 Nov 9;26(22):10878. doi: 10.3390/ijms262210878 (PMC12652428; doi:10.3390/ijms262210878)
Supplement: Supplementary file 1 [file ijms-26-10878-s001.zip › Supplementary File S1 (a) Chromatogram of CR5 at 210 nm (purity 98.5%)..pdf]

Default Individual Report

SAMPLE INFORMATION

Sample Name:	CR5	Acquired By:	System
Sample Type:	Unknown	Sample Set Name:	PEPTIDE
Vial:	1	Acq. Method Set:	peptide_210_230
Injection #:	1	Processing Method:	CR5_210nm
Injection Volume:	100.00 ul	Channel Name:	2998 Ch1 210nm@1.2nm
Run Time:	45.0 Minutes	Proc. Chnl. Descr.:	2998 Ch1 210nm@1.2nm
Date Acquired:	9/18/2025 2:15:42 PM KST		
Date Processed:	9/18/2025 3:09:44 PM KST		

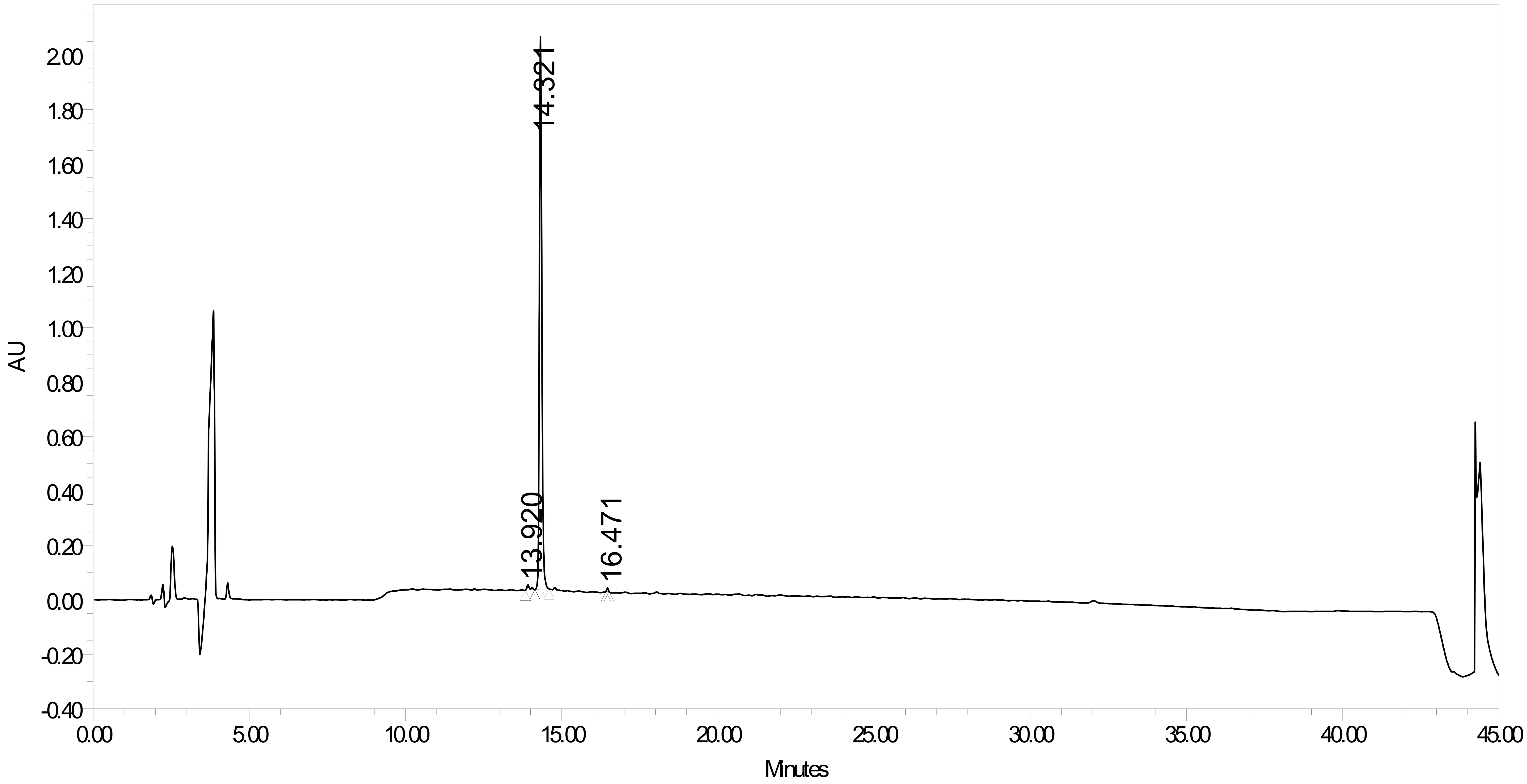

	RT	Area	% Area	Height
1	13.920	119605	1.07	18696
2	14.321	10988493	98.54	2028405
3	16.471	42896	0.38	12794
