# Supplementary material for: In Vitro and In Silico Evaluation of a Novel Multifunctional Cyclic Peptide with Antioxidant, Tyrosinase-Inhibitory, and Extracellular Matrix-Modulating Activities
Source: Int J Mol Sci. 2025 Nov 9;26(22):10878. doi: 10.3390/ijms262210878 (PMC12652428; doi:10.3390/ijms262210878)
Supplement: Supplementary file 1 [file ijms-26-10878-s001.zip › Supporting Information.pdf]

Supplementary File S1. High-performance liquid chromatography (HPLC) peak area integration report (PDF).
(a) Chromatogram of CR5 at 210 nm (purity 98.5%).
(b) Chromatogram of CR5 at 230 nm (purity 98.7%).
Peak area information is included in each chromatogram. The acquisition conditions were identical to those described in Table 1 and Table 2 of the main manuscript.

Supplementary File S2. Exported chromatogram XY data (time vs intensity) obtained from HPLC analysis. X–axis represents retention time (min) and Y–axis represents detector intensity (mAU).(Excel). (a) 210 nm data table (CR5). (b) 230 nm data table (CR5).

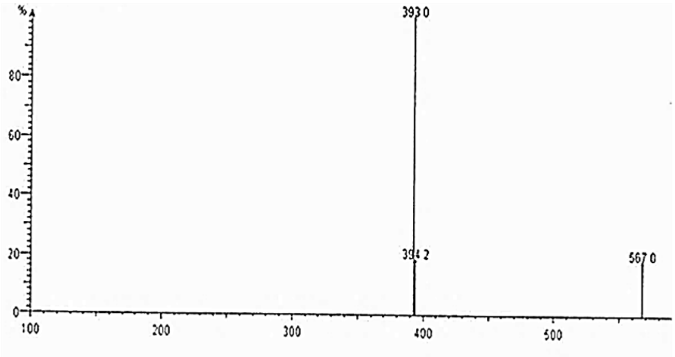

Supplementary File S3. MALDI-TOF MS original graph of CR5.

Supplementary File S4. Cell viability according to CR5 treatment concentration water-soluble tetrazolium salt-1 (WST-1) assay. (a) Cell viability evaluation of CR5 in CCD-986Sk cells. (b) Cell viability evaluation of CR5 in RAW 264.7cells.
The 450 nm absorbance value was converted to zero (%), and the average value and Stdev were presented.
